# Women’s Experiences of Gender-Based Interpersonal Violence in Sport: A Qualitative Meta-Synthesis

**DOI:** 10.1177/15248380241244397

**Published:** 2024-04-09

**Authors:** Kirsty Forsdike, Fiona Giles

**Affiliations:** 1 La Trobe University, Melbourne, VIC, Australia; 2 University of Melbourne, VIC, Australia

**Keywords:** women, sport, gender-based violence, qualitative meta-synthesis, feminist socio-ecological model

## Abstract

Violence against women in sport is pervasive. Prevalence rates of interpersonal violence range from 26% to 74% across psychological, physical, and sexual violence. This review synthesizes adult women’s experiences of gender-based interpersonal violence in sport. A systematic review of qualitative studies was conducted. Five databases were searched, including CINAHL, Web of Science, SPORTDiscus, PsycINFO, and Sociological Abstracts. In total, 1,617 records were retrieved and screened. Twenty-five records representing 24 studies were eligible for inclusion. Following a meta-ethnographic approach, both authors synthesized first- (participants) and second-(researcher) order constructs to create a new interpretation (third-order construct) beyond the individual studies reviewed. A feminist socio-ecological lens was applied. Five themes were constructed: women’s safety work, the normalization of abusive behaviors in the sports context, sport family violence, organizational impotence and hostility, and women’s status in a patriarchal system. Women’s experiences of abuse are mapped within and across the individual, relational, organizational, and cultural levels of the socio-ecological model, with (lack of) power being a central factor within each level as well as flowing between the levels. A fifth socio-ecological level was developed pertaining to the unique context of sport—that of the sport family. This sits between the relational and organizational levels of the model and covers both intense familial relationships and patriarchal familial organizational structures in sport that facilitate and silence the abuse. Sporting bodies must co-design interventions encompassing all socio-ecological levels to address gender-based violence in sport.

## Introduction

This qualitative meta-synthesis, taking a meta-ethnographic approach, reviews the empirical qualitative literature on adult women’s experiences of gender-based violence in sport. Violence against women in sport is a pervasive issue. Prevalence rates of interpersonal violence against women range from 26% to 74% across psychological, physical, and sexual violence (Ohlert et al., 2021). This is despite sport being championed as a site for women’s empowerment and equality, and as a key setting for addressing the drivers of gender-based violence through prevention programs (Hayhurst et al., 2018; Jaime et al., 2015; Our Watch, 2021). While prevalence studies show a high rate of interpersonal violence, they often focus on sexual violence, or elite and youth sport populations (Forsdike & O’Sullivan, 2022). This qualitative meta-synthesis provides greater insight to broader experiences across age, type of violence, and includes non-elite sport participation.

For this meta-synthesis, we were acutely aware of the challenges with definitions in this space. With regard to gender-based violence, we were guided by the UN’s definition of violence against women in Articles One and Two of the Declaration on the Elimination of Violence against Women (Proclaimed by General Assembly resolution 48/104 of December 20, 1993): “any act of gender-based violence that results in, or is likely to result in, physical, sexual or psychological harm or suffering to women, including threats of such acts, coercion or arbitrary deprivation of liberty, whether occurring in public or in private life” (Article 1). The sport academic literature over the last 30 years has used a variety of definitions for forms of interpersonal violence, relating to all genders, most often using sexual abuse. In 2007, the International Olympic Committee’s consensus statement used the terms “sexual harassment and abuse,” updating it to “non-accidental violence” in 2016 to include broader definitions of violence, including psychological and physical violence as well as neglect (Ljungqvist et al., 2008; Mountjoy et al., 2016). By using the UN definition, we aim to be inclusive of all forms of violence, including both interpersonal and violence potentially perpetrated or facilitated by institutions.

Sport is no easier to define. It is a nuanced concept that draws upon the immediate activity as an individual or group, the cultural and societal context in which it is engaged, as well as having a social relational dimension (Lagaert & Roose, 2016). Sport can be viewed as specific activity that is structured and competitive (Giulianotti, 2005) but can also encompass “engagement” that includes facilitating and spectating (Nicholson & Hoye, 2008). Sport has also long been seen as a gendered, patriarchal, social institution with “quasi-religious rituals” (Giulianotti, 2005, p. xv), that exemplifies dominant hegemonic masculinity, and associated violence (Clark, 2017; Messner, 1992). In this context, the connection with between the culture of sport and violence against women is well recognized (Forsdike et al., 2022).

The impact of gender-based violence across broader society is well known, negatively affecting the physical and mental well-being of women and children (Lum On et al., 2016; Savopoulos et al., 2022). In sport, it has been less researched particularly among adult women, but it is clear that girls who have experienced violence in sport report poorer mental and physical health outcomes (Daignault et al., 2023; Parent et al., 2022; Vertommen et al., 2018). The rates of violence against women in sport are high and include psychological, physical, and sexual violence (Lang et al., 2022; Ohlert et al., 2021). The rates of prevalence across gender-based violence in sport are broad, for example rates of sexual violence have ranged from 0.5% through to 78%, depending upon whether studies have included sexual harassment and/or sexual contact only, or particular perpetrator types, for example coaches (Fasting et al., 2003; Parent et al., 2016). While men and boys also experience such violence, it is clear women and girls experience certain types of violence more than men and boys, with all more likely to experience such violence from men (Lang et al., 2022; Vertommen et al., 2017).

Although there has been increased focus on the issue of interpersonal violence in sport in recent years, following three decades of foundational work by Celia Brackenridge, Kari Fasting, and Sandra Kirby, there is no contemporary literature review that synthesizes the literature on women’s experience of gender-based violence. We address this by conducting a qualitative meta-synthesis so that women’s collective voices can provide an enriched, coherent system of knowledge about the topic (Major & Savin-Baden, 2011). Additionally, tested response and prevention initiatives appear to still be in their infancy (Forsdike & O’Sullivan, 2022; Hartmann-Tews et al., 2020). Many of the existing policies, practices, and procedures used today, across national sports organizations or government entities, have not been evaluated or their evaluations are not easily available (Rulofs & Wagner, 2018; Vertommen et al., 2015). However, there is consensus that any initiative development in the future must be collaboratively co-designed with those it intends to protect and ensure victim–survivor voices are heard (Mountjoy et al., 2022; Rulofs et al., 2019; Tuakli-Wosornu & Kirby, 2022). To this end, we report on that a meta-synthesis that explored women’s experiences of gender-based violence in sport so that it may inform future initiative development. Thus, the research questions for this study are: (1) What are adult women’s lived experiences of gender-based violence in sport? (2) How do the studies examined in this meta-synthesis and their findings relate to one another and their varying contexts?; (3) How can the findings be developed into a model that can inform policy and practice?

## Methods

This qualitative meta-synthesis was informed by Noblit and Hare’s guidance (1988). The synthesis involved several stages: (1) developing a research question, (2) conducting a systematic search of the evidence, (3) screening studies according to inclusion and exclusion criteria, (4) conducting a quality assessment of each study using a standard template, (5) extracting key information about each study using a standard template, (6) extracting first- and second-order data; and (7) conducting thematic analysis. We describe these stages in further detail below.

### Search Strategy

Two database searches were conducted, the first in November 2020 with no time limit applied (search 1), and repeated in June 2022 for the time period December 2020 to May 2022 (search 2). Five databases were searched: CINAHL, Web of Science, SPORTDiscus, PsycINFO, and Sociological Abstracts. The search strategy was designed in the EBSCO platform then tailored using the specific indexing language of the other platforms relevant to the databases being searched. Four categories of search terms were combined: (1) gender-based violence/abuse/harassment, (2) sport/exercise, (3) woman/female, and (4) qualitative. Keywords and MeSH terms were both used. The detailed search strategy can be referred to in Supplemental Appendix A.

### Selection of Studies

Search results were initially imported into Endnote and then into Covidence, software that assists researchers to manage systematic reviews (Covidence systematic review software, 2022). Article title and abstract screening was conducted by the second author with potentially eligible studies included for full-text review. Inclusion criteria were: English language only; primary studies that used a qualitative design or mixed methods studies where qualitative findings could be separated out from quantitative findings; presence of direct participant quotes; adult women describing their experiences of any gender-based violence; and women actively participating in sport as athlete, coach, official, or volunteer. Exclusion criteria were: findings about gendered violence against women could not be distinguished from other types of abuse (e.g., non-gendered child abuse); case descriptions with no systematic interpretation of data; participants worked in the non-sporting context (e.g., media, health, education); participants were not actively involved in the sport (e.g., fans); and e-sports (i.e., online gaming, fantasy sports).

Both authors carried out full-text screening by applying the same inclusion and exclusion criteria. In determining whether women were “adult,” the reported age of the majority of participants in reviewed studies could not be under the ages of 16 years of age. Disagreements were reached by consensus where possible, with a third researcher available to advise and resolve disagreements that were unable to be resolved by consensus. For one article where there was uncertainty, the first author contacted the article’s primary author for clarification.

### Quality Appraisal

Study quality was assessed using a Critical Appraisals Skills Program (CASP) tool for qualitative research (Critical Appraisal Skills Programme, 2018). Both authors independently appraised the quality of all included studies, then met to reach consensus. This process was undertaken using Covidence. The quality appraisal results did not determine study inclusion; however, they informed the evaluation and interpretation of study findings.

### Data Extraction

A standardized form was developed in Covidence and used to extract study characteristics including research aim(s), location, population characteristics, methodology, theoretical framework, funding sources, researcher positioning, study design, violence type(s) and definitions, ethical considerations, and evidence of trauma-informed practice. Both authors independently extracted these data, then met to reach consensus. Articles were then imported to NVivo, software that enables text to be coded and extracted (QSR International Pty Ltd, 2020). Data were coded according to the first-order constructs of direct participant quotes, paraphrase, and description; or second-order constructs of theme headings and author interpretation. Data were extracted and organized in these groupings for analysis.

### Conceptual Framework

We used a feminist socio-ecological model as a lens through which to analyze and guide interpretation of and synthesize the included literature. The socio-ecological model views human experience as complex and multi-leveled, with “multi-person systems of interaction” across the individual, interpersonal, organizational, and socio-cultural environments (Bronfenbrenner, 1977, p. 514). Since its introduction, the socio-ecological model has been adapted in gender-based violence research to acknowledge that such violence requires a multi-level approach (Heise, 1998), and expanded to take into account global ideologies such as cross-country influences on the experience of gender-based violence (Fulu & Miedema, 2015). A feminist socio-ecological model highlights gender power relations, as they interconnect throughout the socio-ecological levels (LaVoi, 2016), as well as the socio-material-cultural practices that exist across sport, such as the interplay between the individual experience and local, national, and global safeguarding policies (Aitchison, 2005). The model is well suited for a meta-synthesis of women’s experiences as it examines the interplay between the individual experience, relationships with the perpetrator and closely connected sport, family and peer communities, and broader contexts, such as sport cultures and institutional processes.

### Data Analysis

We used reflexive thematic analysis to explore, examine, and interpret the data (Braun & Clarke, 2022). Taking a meta-ethnographic approach enables the construction of an interpretation of the collective of qualitative studies rather than adding together the individual data components from each study. The ethnographic component then sees the translation of studies into one another, moving back and forth between them as an interpretation of the collective is constructed (Noblit & Hare, 1988).

Both authors immersed themselves by reading and re-reading the extracted data both in Nvivo and in printed copies of the data. Initial descriptive codes were generated based on the first- and second-order data extracted from the articles. Interpretative codes were then created that represented deeper layers of meaning. Finally, these interpretative codes were grouped to represent common themes across the dataset. This process occurred iteratively and flexibly, where codes and themes were discussed frequently by the two authors until consensus was reached on the groupings and interpretation of themes and codes. The themes and sub-themes were mapped against the feminist socio-ecological model’s existing four levels: individual, interpersonal, organizational, and socio-cultural environments.

### Researcher Reflexivity, Care for Ourselves, and Care for Article Readers

Both authors have participated in various forms of sport at different levels and in varying capacities since our early 20s. We have both experienced gender-based interpersonal violence through our engagement with sport. One author is also a survivor of intimate partner violence. Before commencing this qualitative meta-synthesis, we established trauma-informed protocols to minimize the risk of vicarious trauma from conducting research on this topic. This included weekly meetings to “check-in” with each other, a reflective journal, limiting the number of articles reviewed per day, and taking breaks when the content of the articles being reviewed was causing personal distress. The ethnographic approach proved challenging. Reading and re-reading traumatic stories, comparing, and translating these into other traumatic stories on a regular basis took its toll emotionally. Discussions of the articles invariably brought up discussions of our personal experiences and how these played a part in our individual interpretations. The trauma-informed protocols that we set up were regularly reflected upon and adjusted as the need for self-care became increasingly evident.

This article contains descriptions and direct quotes from study participants that may be confronting and distressing to read. We encourage you to consider your position and experiences in relation to the content and plan self-care strategies accordingly.

## Results

### Overview of Studies

**Figure 1.**
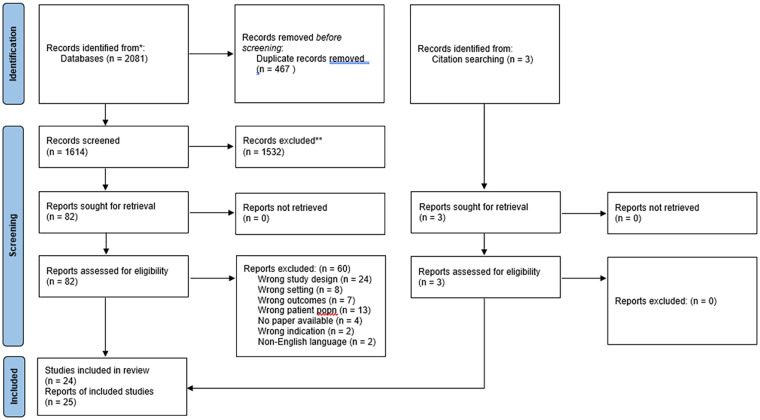
PRISMA diagram. Two searches were conducted. Search 1: (no earliest time limit)—November 2020; Search 2: December 2020–May 2022.

The database searches generated 1,756 (search 1) and 325 (search 2) records. After duplicates were removed, 1,383 (search 1) and 234^1^ (search 2) records remained. The title/abstract screening generated 86 records for full-text screening. Following full-text screening, 25 records were eligible for inclusion. Two records were reporting the same data (one was a book; the other was an article containing material from a chapter in the book); these records were combined. Other records were reporting data from the same research project; however, as each article addressed a different research question; these were kept as separate records. Therefore, 24 studies representing 25 records were included in the final analysis (Figure 1). The CASP checklist of studies and the detailed justification for each response is provided in Supplemental Appendix B. The CASP showed quality of studies to be mixed for methodological rigor.

### Study Characteristics

Table 1 below summarizes the studies included in this meta-synthesis. The detailed characteristics of each of the 24 included studies are presented in Supplemental Appendix C. The methods and methodologies used for each study are presented in Supplemental Appendix D. Papers were published between 1997 and 2022, with 17 published since 2010 and a large number (10) published in the last 2 years. Most of the studies were conducted in Western countries, with Canada (5) and Norway (4) producing the most published studies. There were four (4) studies conducted across multiple countries, including a systematic review, and one (1) study where the country was not disclosed to protect the identity of the participant.

**Table 1.** Included Studies’ Characteristics.

Study Characteristic	No. of Studies
Year of publication^a^	
1990–1999	2
2000–2009	6
2010–2019	7
2020–2022	10
Participant role^b^	
Athlete, current and/or former	22
Coach	1
Umpire/official	1
Manager/administrator	2
Primary type of violence specified	
Sexual	22
Physical	9
Psychological	6
Technology facilitated abuse	3
Financial	2
Perpetrator	
Coach	17
Authority figure (Dr, masseuse, dietician, manager)	4
Peer^c^	6
Member of public (e.g., fans, strangers)	6
Family member^d^	1

a This is the only characteristic to report groupings by publication (*n* = 25). All others report groupings by studies (*n* = 24).

b Studies may be counted more than once due to participants having multiple roles for example, former athletes who stay involved in sport.

c Usually other athletes.

d Perpetrators were both male and female.

With respect to diversity, 13 studies did not report on the ethnicity of the participants, and only two documented disability status (one of which was a study about paralympic athletes). For studies with a small number of participants, study author(s) did not always detail the diversity of the participants due to participant confidentiality. The age of participants, including the age at which the abuse occurred, was not specified for six studies, and as with ethnicity, sometimes this was to protect the confidentiality of participant(s). For those studies where ethnicity and disability status were documented, the vast majority of participants were White/Caucasian, followed by Hispanic/Latina and Black/African American.

Most studies (22) were conducted with current or former athletes. Two studies were conducted with sports managers/administrators only, one with umpires/officials only and one with coaches as well as athletes. Perpetrators of gender-based violence in sport were primarily reported to be coaches (17), followed by peers (6), members of the public (6), other sports authority figures (4), and family members (1). In studies with large samples, perpetrators were across some or all these groups.

### Forms of Violence

Table 2 summarizes the forms of violence experienced by the study participants and includes examples of the language used to describe it. Sexual harassment and abuse were the most common form of gender-based violence reported in the studies we examined. Included studies provided examples of women who had reported experiencing sexual harassment by multiple perpetrators; women who were victim survivors of intimate partner violence and attempted homicide by their coach, women who were sexually abused by the team doctor; women who experienced harassment by members of the public and women who had been groomed and sexually abused by their coaches when they were teenagers, then experienced intimate partner violence with the same perpetrator when they became adults. Women who were abused over a prolonged period often experienced multiple forms of violence (sexual, physical, emotional, and financial), which intersected and overlapped.

**Table 2.** Forms of Violence.

Type of Violence	Examples	Studies
Sexual abuse, grooming, harassment	Rape, non-consensual touching, secret meetings and communication, making the athlete feel special, cat calls, name calling (slut, bitch, pussy), wolf-whistles, sexually explicit comments, sexist jokes, exposure to porn, unwanted sexual advances, forced lap sitting	Alexander et al. (2020), Armstrong and Hutchison (2021), Bisgaard and Stockel (2019), Brackenridge (1997), Brockschmidt and Wadey (2022), Fasting et al. (2002), Fasting et al. (2007), Fasting and Brackenridge (2009), Fasting and Sand (2015), Hayden (2004), Hussain et al. (2021), Kirby et al. (2000), Klavenes et al. (2020), Krauchek and Ranson (1999), Owton (2016), Owton and Sparkes (2017), Rodriguez and Gill (2011), Stirling and Kerr (2009), Tamminen et al. (2013), Tingle et al. (2014), Vasudevan and Ford (2022), Way (2023)
Physical assault, body shaming, forced training/dieting	Stabbed, pistol-whipped, bashed, slapped, punched, objects thrown at head and body, told they were flat chested, shaped like a pear, had a big bum, looked like a man, disgusting, fat pig, excessing forced training, severe calorie restricted diet, forced drug use	Alexander et al. (2020), Hussain et al. (2021), McMahon and McGannon (2020), McMahon et al. (2021), Stirling and Kerr (2009), Van Ingen (2021), Vasudevan and Ford (2022)
Psychological coercion, control, isolation, emotional abuse	Stalked, threatened with future violent attacks, gaslighted, intimidated, manipulated, humiliated, degraded, yelled at, encouraged/forced to use illicit drugs	McMahon and McGannon (2020), McMahon et al. (2021), Owton (2016), Stirling and Kerr (2009), Van Ingen (2021), Way (2021)
Financial	Restricted access to work, having to ask for money	Owton (2016), Van Ingen (2021)
Technology facilitated abuse	Text messages, obscene phone calls, revenge porn	Bisgaard and Stockel (2019), Kirby et al. (2000), Van Ingen (2021)

### Impact of Gendered Violence

Thirteen studies reported the impact of gendered violence on victim survivors, albeit some doing so only briefly. Participants reported a range of emotional responses including “shame,” “isolation,” a sense of “helplessness” and “hopelessness”; many blamed themselves for their own abuse and for the perpetrator’s continued abuse of other athletes (Brockschmidt & Wadey, 2022; Fasting et al., 2007; Kirby et al., 2000; Tamminen et al., 2013). Participants reported experiencing anxiety, depression, and post-traumatic stress disorder (Kirby et al., 2000; Owton, 2016). Some participants also expressed “anger” and “disgust” toward the perpetrator of their abuse, and/or at the poor organizational response (Brockschmit & Wadey, 2022; Fasting & Sand, 2015; Fasting et al., 2002, 2007). For some women, these emotional responses only emerged several years after their experiences (Kirby et al., 2000). One participant engaged in risky sexual behavior as a way of “self-managing” her abuse and was subsequently retraumatized (McMahon & McGannon, 2020).

Participants also described feeling “fearful” of direct recriminations by the perpetrator, of others finding out about the abuse, and of the impact on their sporting career (Fasting et al., 2007; Kirby et al., 2000; Stirling & Kerr, 2009). Many of these fears were realized, with participants describing that after they rejected sexual advances or reported sexual harassment or assault, they lost their place on the team, or were subject to unfair treatment by the perpetrator and other members of the sporting family (Way, 2023). In the case of Larry Nassar, athletes who tried to voice concerns were mocked in front of others and felt humiliated (Way, 2023). Many participants reported they had to change coach, change sports, or drop out of sport entirely (Bisgaard & Stockel, 2019; Fasting et al., 2002; Rodriguez & Gill, 2011). Conversely, some participants experienced “ambivalent” or “mixed” feelings about their perpetrator (usually the coach) (Brackenridge, 1997). Participants who had successfully sought justice via the legal system or who had received extensive therapeutic treatment described a process of a “reclaiming of self” and a strong motivation to help others and prevent future gender-based violence in sport (Owton, 2016).

### Themes

Our analysis of the first- and second-order constructs generated five overarching themes relating to women’s experiences of gender-based violence in sport. Four of these themes mapped against the feminist socio-ecological model, these were: women’s safety work; the normalization of abusive behaviors; organizational impotence and malevolence; and women’s status in a patriarchal system. We identified a fifth theme specific to the sport context, that of sport family violence, which sits as a new socio-ecological model level between the interpersonal and organizational levels. Table 3 below summarizes each theme, referencing the relevant literature.

**Table 3.** Summary of Themes.

Theme and Sub-Themes	Summary	Literature
Women’s safety work • Behavior modification or avoidance • Collective action	The response to abuse is women’s responsibility, with women working to keep themselves Safe.	Armstrong and Hutchison (2021), Bisgaard and Stockel (2019), Brockschmidt and Wadey (2022), Fasting and Brackenridge (2009), Fasting and Sand (2015), Fasting et al. (2002), Fasting et al. (2007), Kirby et al. (2000), Klavenes et al. (2020), Rodriguez and Gill (2011), Armstrong and Hutchison (2021)
Normalization of abusive behaviors • Comes with the territory • Desensitization	Abuse comes with the territory of elite athlete and competitive success, where behaviors are excused to get results.	Alexander et al. (2020), Armstrong and Hutchison (2021), Fasting and Brackenridge (2009), Fasting et al. (2007), Kirby et al. (2000), Klavenes et al. (2020), Krauchek and Ranson (1999), Rodriguez and Gill (2011), Stirling and Kerr (2009), Tingle et al. (2014), Way (2023)
Sport family violence • Surrogate family • Omniscient father figure	The closeness of the relationships within sport mirror familial relationships, built on dependencies and trust that is abused. The omniscient father figure of the coach is feared and respected, idolized and powerful.	Bisgaard and Stockel (2019), Brackenridge (1997), Fasting et al. (2007), Kirby et al. (2000), Owton (2016), Owton and Sparkes (2017), Stirling and Kerr (2009), van Ingen (2021), Way (2023)
Organizational impotence and malevolence • Organizational impotence • Organizational malevolence	Sports organizations create an unsafe environment either through passivity or organized intentional cruelty where success overrides safety.	Brackenridge (1997), Fasting et al. (2007), Rodriguez and Gill (2011), Stirling and Kerr (2009), Way (2023)
Women’s status in a patriarchal system • Women as “other” • Boys club	Women are seen as inferior to patriarchal hierarchy and treated as “other.” Men engage other men as enablers and supporters of violence.	Fasting and Brackenridge (2009), Hussain et al. (2021), Kirby et al. (2000), Klavenes et al. (2020), Krauchek and Ranson (1999), Rodriguez and Gill (2011), Tingle et al. (2014), van Ingen (2021), Vasudevan and Ford (2022)

#### Women’s Safety Work

At the individual level, the response to interpersonal violence in sport was seen as women’s responsibility. Quotes from women participants across the studies, and the authors’ interpretations, showed that there is extensive safety work being undertaken by individuals in the face of gender-based violence in sport. This was a particular theme constructed by Armstrong and Hutchison (2021) in their study of women’s sport participation in New Zealand, but which also resonated across multiple studies.

There were two subthemes within Women’s Safety Work: behavior modification including avoidance; and collective action. Behavior modification was clearly apparent and referred to by Brockschmidt and Wadey (2022) in their study of harassment while running in London as the “cost of safety” (p. 594). It speaks to the work women do to keep themselves safe, with avoidance being the most oft reported labor undertaken. Such avoidance behavior followed a spectrum from small acts of physical avoidance to significant life-changing acts that included leaving sport altogether:

I try to have as little to do with him as possible. . . and avoid being alone with him (Fasting et al., 2002, p. 11).

I stopped running in winter/after 6pm and joined a gym to run on a boring treadmill instead. If I am not running with friends at night, I avoid it and save it for daytime runs on the weekend instead of after work (Brockschmidt & Wadey, 2022, p. 354).

Three of the six athletes made drastic decisions in response to their sexual harassment encounters. Two athletes dropped out of their sport and one withdrew from the team without returning in future years (Rodriguez & Gill, 2011, p. 11).

Other forms of behavior modification were either reactively or proactively taken. These behaviors could relate to reactively removing their bodies from immediately unsafe spaces or actions such as “avoiding massages, avoiding exposing their body” (Rodriguez & Gill, 2011, p. 14), and “keeping a 2 m distance from” the perpetrator (Fasting et al., 2007, p. 427). Proactive self-safety behaviors could involve simple acts such as choosing alternative clothing as a means of protecting from the male gaze but also strategically planning where to train to enhance safety:

One woman also said she felt that she had to dress formally and discreetly at work to avoid unprofessional looks from her male associates (Klavenes et al., 2020, p. 357).

I don’t think you have the option just to train anywhere you like. You’ve got to be cautious of that . . . like depending on where I’m training, I might wear different clothes. So, if I know that there are . . . men that are going to be in this area that I have to pass I will definitely wear looser clothing, or I’ll try not to draw attention to myself (Armstrong & Hutchison, 2021, p. 13).

Further behavior modification involved a significant amount of planning, often enlisting the help of others. For example, in relation to street harassment when running, women participants described their safety behaviors as:

including letting someone know where they are going and when they will be back, running with their dog or a training partner, running without headphones to be more alert of potential dangers, carrying their mobile phone, or carrying their keys as a makeshift weapon (Brockschmidt & Wadey, 2022, p. 354).

Some participants across the studies described safety work that involved active resistance or confronting violent behaviors, although this often required collective rather than individual action. At the individual level, women saw their safety work as preparing to fight:

I became afraid of going out alone. . . I started to have a knife in my pocket when I was going out. . . I also started practising karate at that time. (Fasting et al., 2002, p. 11).

Listen, [my sport] was the first combat sport that the [main] sport agency included in international competitions. It was a male space. . . [at that time] we were not little girls, we confronted them and they didn’t like it. I remember that I was always fighting in and outside sport. (Rodriguez & Gill, 2011, p. 10)

Yet, it was clear that to change the unsafe environment, women needed to work as a collective to voice their experiences, seek justice and confront abusers:

After a while, I talked with one of the other parents. . . She helped me and the other girls to bring our cases forward. . . The trial lasted for two-and-a-half years (Bisgaard & Stockel, 2019, p. 235).

My teammates were older than me . . . they advised me and whenever they protested [against the harasser], I followed their lead and protested also. I had the advantage to count on someone older than me and with more experience (Rodriguez & Gill, 2011, p. 13–14).

#### Normalization

Normalization of abusive behaviors was a strong theme running throughout the first- and second-order constructs across almost all the studies included in the review. Abusive behaviors, particularly of male coaches, are viewed as expected in the sport context and are excused in order to get results. Equally, gender-based violence is perceived as a normal part of a woman’s life, and as such women become desensitized to it.

The women in the studies included in the review talked about the abuse they experienced or witnessed as a normal part of their lives: “Alex, for example, felt it was inevitable that young women would be harassed when taking part in sports” (Armstrong & Hutchison, 2021, p. 13). This sense of normalcy would sometimes evolve into a desensitization and ambivalence toward abusive behavior:

If they (men) address me with offensive comments, I believe these men to be very ignorant people. I do not take it personally (Klavenes et al., 2020, p. 356).

Women in the studies sometimes talked in ways that appeared to indicate that they did not recognize the behaviors as abuse even as they told their stories to the researchers with some acknowledging that they did not realize the behavior was abusive until later in life.

However, it was the sport context that seemed to elevate the apparent abusive behaviors to being an expected part of male dominance in sport, with “many coaches” seen as “male predators” (Krauchek & Ranson, 1999, p. 592):

My coach commented on our body and slapped my ass and stuff like that all the time. It’s a lot like that in our sport (Alexander et al., 2020, p. 54).

“When there’s a bunch or 16-to 20-year-old girls who are quite athletic you know you’re always going to have the odd creepy guy around.” She reflected, “I’m not saying that’s how it should be but that’s how it is.” (Armstrong & Hutchison, 2021, p. 13)

Such abusive behaviors were reported as an accepted part of playing sport, particularly in elite sport and competitive environments, or where spectators are present:

It was his way of behaving, his type of humour and style . . . he wanted us to look physically like top level athletes, he was very occupied with our appearance, but sometimes it was difficult to separate what he was saying to us as athletes and what he was commenting about us as women” (Fasting & Brackenridge, 2009, p. 27)

Expected settings (e.g., competitions) are those places in which sexual harassment sometimes is tolerated. Participants mentioned that sexual approaches were expected particularly from male spectators and individuals that were visibly harassing other female athletes. (Rodriguez & Gill, 2011, p. 9–10).

#### Sporting Family Violence

The sport family theme brings together the view of sport and the closeness of the relationships within it as a surrogate family, with an omniscient father figure of the coach. The coach is both feared and respected, idolized and powerful.

The coach as a father figure was a consistent theme across several studies. From athletes being referred to as “Doug’s little girls” (Kirby et al., 2000, p. 22) through to recognition that the time spent in the sport family unit was often more than with their own families:

The participants spent a significant amount of time with their coach and compared their relationships to that of a father-daughter. . . relationship. Alexandra explained that as she grew up she spent more time with her coach than with her own family. Likewise Caitlyn said, “We saw our coach more than we saw our parents. It sounds kind of weird but he was like a quasi-father ﬁgure . . . He probably knew stuff about some of us that our parents didn’t even know” (Stirling & Kerr, 2009, p. 231).

This was the bloke I’d trusted . . . he knew everything about me—more than my parents (Brackenridge, 1997, p. 123).

In particular, the term “father figure” is repeated several times across Owton’s (2016, 2017) book and article reporting Bella’s experience of sexual abuse to describe the coach’s relationship with her. Given the loss of her father at a young age, the coach taking this position has even greater significance. Owton (2016) details the situation Bella experienced and abuse that arose as evolving into athlete domestic violence, which, as Owton explains, is referred outside of the sport context as intimate partner violence or abuse (p. 56). Having this terminology and connection to intimate partner violence, Owton aligns Bella’s experience with the Duluth Model of Domestic Violence. However, family violence would also be a useful term to consider given the situation of the team as family, the coach as a father figure and Bella as both a child within that surrogate family as well as intimate partner of the coach, also her father figure.

As with intimate partner violence and family violence, isolation is used to separate women being abused from both the sport family and their actual family together with coercive control to maintain an environment of secrecy and dominance:

Anna talked about her coach preventing her from talking to her mom about his behaviours. “He [coach] told me I wasn’t allowed to call home to talk because he didn’t want me to tell my mom anything he said. So he was very controlling in that way as well” (Stirling & Kerr, 2009, p. 234).

The elite athlete will often spend more time with coaches than with parents or friends, which may lead to an emotional dependency. She may think that he or she is the only person who can help her to greater athletic success. As a result, she may tolerate more from the coach than she does from other adults in her surroundings (Fasting et al., 2007, p. 425).

He started to behave different towards me during practice. It was like he punished me. Without any reason he put me on another team at a lower level. This also meant that I was separated from my friends during practice (Bisgaard & Stockel, 2019, p. 232).

Intentionally kept from their homes and their support systems, girls were “worked into exhaustion by screaming coaches” (Way, 2021, p. 13).

The coach or sports doctor as father figure, omniscient patriarchal power demands control and isolates those beneath him from broader supports:

For Victim 10, meeting Nassar, “who carried off [her] idol, Kerri Strug, only a couple years before” for an assessment of her injury, was “equivalent to meeting a celebrity” (Way, 2021, p. 10).

Because he coaches the best people in the country no-one questions him (Brackenridge, 1997, p. 120).

There are a few other lads around—they all dutifully follow Ray’s lead, including myself who look up to him as my instructor, coach and a father figure. Ray’s not only well respected by his whole team, but when we go to competitions he’s got a tough reputation and seems to know everyone; quite a few are rather scared of him. He’s fearfully respected. (Owton, 2016, p. 27; Owton, 2017, p. 735)

The coach or, in the case of Dr. Larry Nassar, has significant social and cultural capital that protects him—the successful performances attributed to their work hides or glosses over the practices taken.

#### Organizational Impotence and Malevolence

These two organizational characteristics of impotence and malevolence sit at opposite ends of a spectrum, each creating a hostile environment either through passivity or organized intentional cruelty.

Impotency sees a powerlessness or weakness to act. This includes inadequate responses, non-existent policies, or inappropriate implementation of policies. Complaints go nowhere and there is a lack of oversight or accountability:

When the athletes were interviewed the Norwegian Sport Organizations had no policy for handling sexual harassment. Sexual harassment was more or less a non-issue for these organizations at that time. This situation meant, for example, that no code of conduct for athletes, coaches, or sport administrators existed. In addition, the organizations had no procedures for managing sexual harassment cases (Fasting et al., 2007, p. 429).

She reported the abuse but the (sport organization) didn’t know how to deal with it (Brackenridge, 1997, p. 120).

Athletes indicated that the apparent reason for the poor confidentiality is the absence of formal procedures to file sexual harassment complaints and that “everyone knows everyone” in the sport setting. The common phrase “everyone will know” presents a major dilemma for this small island. The apparent administrative negligence in dealing with the complaints has provoked pessimistic feelings among these former athletes (Rodriguez & Gill, 2011, p. 11–12).

Impotence of organizations moves toward malevolence when policies exist but are ignorantly or actively ignored. Fasting et al. (2007) interpreting their interview data raise the lack of policy as an issue. However, this abuse can happen when policies do exist. It is not the presence of a policy that is key. It is the appropriate enactment of the policies into practice. Inappropriate enactment of existing policies can be malevolent action:

Rachael Denhollander’s reports of abuse were mocked by university officials, who told her she “hadn’t really been penetrated. I only thought I had because, quote, when I am a 15-year-old girl I think everything between my legs is my vagina” (Way, 2021, p. 14).

At the other end of the spectrum is institutionally organized abusive responses to women. Hostile environments are created where success overrides safety and athletes are treated as disposable commodities:

Nassar’s abuse was a direct result of the strategies the organization deliberately employed to realize a narrow and extreme version of success. Violence was not the result of one disturbed man, it was built into the institution as a means to success. (In)formal organizational policies and practices played as much of a role as the wayward doctor in structuring violence as a means of achieving organizational goals, while encouraging discourses that concealed and sustained those violent structures (Way, 2021, p. 9).

a coach’s reputation of success was used to justify problematic behaviours (Stirling & Kerr, 2009, p. 232).

She disapproved the sport government’s decision because they avoided taking an objective stand in the process and she believed that the consequences were the result of his power position in her sport. Veronica’s lawsuit took four years to end and she suffered gender discrimination and other forms of psychological stress from peer athletes and other authority figures after the formal complaint. Years have passed since her harassment incident and she compares the complexity in solving a harassment case with “the mafia.” Another athlete affirmed this perspective, explaining that when a harasser has an untouchable power position, the formal accusations vanished leaving the victims without formal evidence (Rodriguez & Gill, 2011, p. 12).

#### Women’s Status

The themes described above all sit within a socio-cultural environment where women’s status is diminished in a masculinized hegemonic space. The theme of women’s status ran throughout the included studies. Here, we see women as inferior to the patriarchal hierarchy and treated as “other.” Those who exist in this space may not even be viewed as a woman (Van Ingen, 2021).

There is a hostility to women, and perceived threat to the hegemonic masculinity of sport. Sport is not seen as a space that women can occupy, with athletes accepting “it is the world of men, it is theirs, to excessive levels. You are doing him a favor by letting you (as a woman) into his world and playing his game” (Klavenes et al., 2020, p. 356):

people cannot accept women’s football because you have to move a lot. . ..and people can see your body (Hussain et al., 2021, p. 9).

“With him it is that he has this opinion about women that they should keep silent and do what they are told. That’s it,” said Kristin. Some athletes were given the feeling that women should not even participate or compete in their sport (Fasting & Brackenridge, 2009, p. 28).

Athletes in non-traditional female sports (e.g., judo) appeared to encounter more severe gender harassment and violence by male authority figures than those in traditional sports (e.g., volleyball) (Rodriguez & Gill, 2011, p. 12).

I’ve had strangers come right up to me in the gym and just say, “You’re a woman, women shouldn’t be muscular. Female bodybuilders look disgusting,” “She looks like a man” (Vasudevan & Ford, 2022, p. 683).

Men engage other men as enablers to maintain the status quo including preventing advancement of women in leadership positions within sport management: “You’ll never move up because you’re a girl and it’s a good old boy network” (Tingle et al., 2014, p. 13). Reinforcing the hierarchy, women are belittled and subordinated, infantilized:

When speaking of an older male official she met at a job try-out, Amber said, “He had tried to work on the men’s side, but now he said he was “settling” for the women’s side” (Tingle et al., 2014, p. 13).

He would say, “I know women, I know athletic women, when you coach them they’re going to cry, they’re going to sulk, they’re wimps, you have to deal with their emotional side” (Krauchek & Ranson, 1999, p. 594).

## Discussion

**Figure 2.**
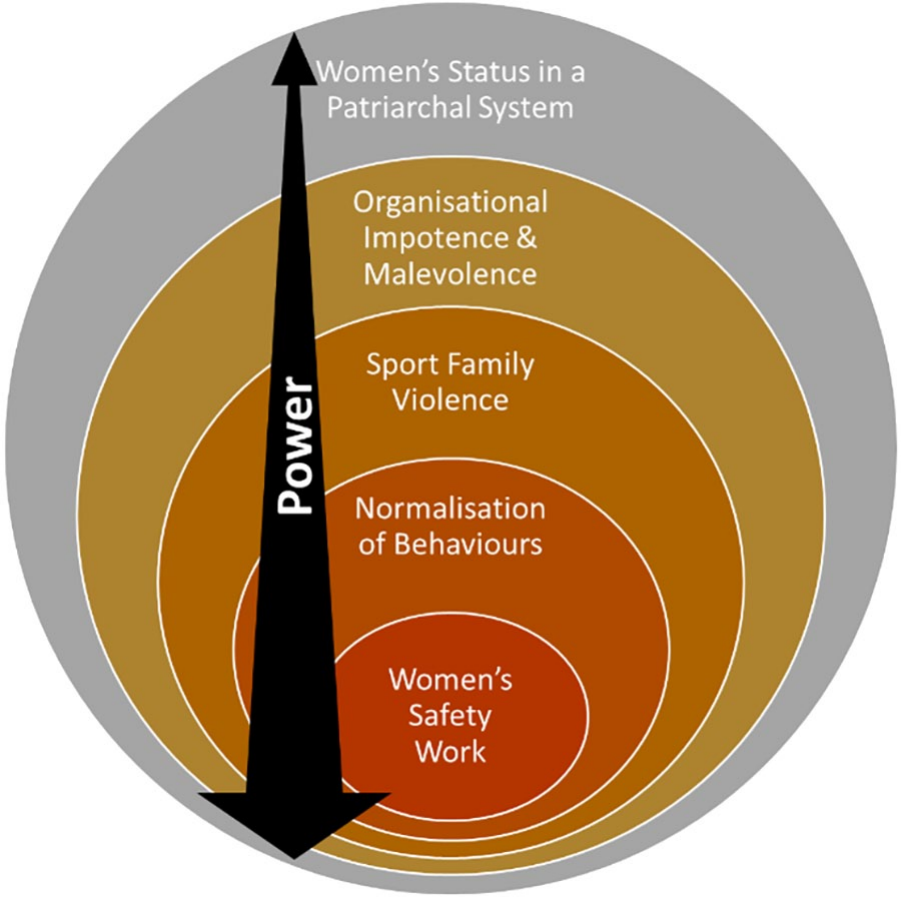
Gender-based violence against women in sport adapted socio-ecological model.

Our qualitative meta-synthesis examined women’s experiences of gender-based violence in sport and identified five themes. We mapped these themes against the feminist socio-ecological model and found alignment for four themes: women’s safety work, normalization, organizational impotence and malevolence, and women’s status. We identified and created a new fifth level, specific to the sport context, that of sport family violence, which sits between the interpersonal and organizational levels. Power, as an underlining undercurrent, facilitates violence and maintains women’s lack of status in sport. It exists within each level and across the model (see Figure 2).

As power being such a dynamic force exerting influence throughout the model, we look to LaVoi’s work on women in coaching and the unequal power imbalances throughout sport that maintain men’s control of and dominance within sport (LaVoi, 2016). LaVoi embedded power as bidirectional throughout the model by drawing upon Foucault and Markula and Pringle’s subsequent application of his work in the sport context. Rather than just a top-down approach to power—women as objects under the power of men—women are seen as subjects within power relations and having the ability to resist (Markula, 2003; Markula & Pringle, 2006). While we agree with LaVoi on bidirectional power throughout the model to some extent, interpersonal gender-based violence in sport as presented by the studies in our review lays bare the issue that resistance toward power at the individual level is not equal to top-down power (as it derives from socio-cultural and organizational or even interpersonal environments). Power from the individual level of the socio-ecological model actually requires collective resistance and, even then, rarely matches power from above. As such, we have adapted the model to reflect the imbalance of power, even as it may flow bidirectionally throughout the model.

At the individual level, the women’s safety theme constructed collectively across the studies reflects similar findings in studies examining women’s experience of gender-based violence in other contexts. For example, a study in Australia exploring women and gender-diverse people’s experiences of sexual violence and harassment on public transport found that women undertook significant work to keep themselves safe while traveling (Ison et al., 2023). In this context, Ison et al. (2023) report that women and gender-diverse people undertake significant planning when needing to travel, including when and where, as well as considering what to wear when traveling. They “remain vigilant” and avoid areas deemed unsafe (Ison et al., 2023, p. 17). Similarly, Fileborn and Hardley (2023) reported on the “safety work” young women and girls engaged in to avoid street harassment as they walked to school, which included planning what to wear and how to physically move. Other contexts where women engage in such safety work in the face of gender-based violence include public places (Lennox, 2022) and universities (Bovill et al., 2022). Lennox (2022) raised an important argument that goes beyond women working to keep themselves physically safe, but that also:

given women’s existence in a system of masculine hegemony in which they are systematically denied equal access to resources, status, and power, a potential encounter with men’s violence threatens women not only with physical endangerment and short-term social judgment, but also with the possibility of lost access to crucial symbolic and material resources (p. 256).

Women’s safety work in sport can potentially also be explored as a means of navigating a masculine hegemonic space where they wield little power. Bovill et al.’s (2022) argument that women “trade freedom for safety” (p. 14) may explain the work of avoidance, where their place within a team or a club is maintained, is more oft spoken about than the work of individual or collective resistance.

The normalization of the abusive behaviors women are faced with in broader society is therefore nothing new. However, our study brings new light onto this phenomenon when we see that the behaviors are part and parcel of sport and where not only individuals but also organizations at best ignore and at worst enable gender-based violence experienced by women. It is useful to consider Catley and Jones (2002) theorizing on violence. Their argument is that “patterned relations of harm” and “ritualised violence” by an institution, rather than singular acts by an individual, are often normalized and therefore legitimized acts. Violence, through impotence or malevolence, is shown by the studies in our review to have become embedded in the structures of sport to the point of legitimization. This is closely tied with the coach or doctor’s individual acts of violence, behaviors that are supported and therefore legitimized and normalized by the institutions of which they are a part. A key aim of a sport organization is to be successful, to win. To win at any cost (violent actions of individual coaches or doctors for example) is therefore legitimized (Stirling, 2009).

The final key outcome of our meta-synthesis is the development of a fifth socio-ecological level in the context of sport. Sport family violence sits at the intersections of interpersonal and organizational levels of the model. Sport, in particular the sport team and sport club, has often been referred to as family (Draper & Coalter, 2016; Forsdike et al., 2019; Tacon, 2021) or extended family (Fletcher, 2020). It is recognized as a context for close relationships, trust, and dependencies (Draper & Coalter, 2016), or as Fletcher (2020) defines it: “by familial I am referring to those relationships where we feel loved, valued and supported by people from outside of our immediate family” (p. 160). Sport research literature shows the sport family as a positive phenomenon. While Draper and Coalter align the sense of family with “an associated sense of safety” (p. 57), family violence can be defined as any threatening, dominating, abusive, or violence behavior that coerces or controls a family member or causes the person experiencing the behavior to feel fear. In fact, the Family Law Act 1975 in Australia deems the following acts as family violence: assault, sexual assault, or sexually abusive behavior; stalking; repeated derogatory taunts; unreasonably denying the family member the financial autonomy; preventing the family member from making or keeping connections with his or her family friends or culture; and unlawfully depriving the family member of their liberty. These behaviors were often mentioned across the studies in this meta-synthesis. If the sport team or club can be considered akin to family, then should we be looking to the family violence sector to understand, identify, respond, and ultimately prevent such violence among the sport family?

## Conclusion

Women in sport often experience composite abuse, intersecting, and overlapping, with power imbalance being at the heart of their experiences. Experiences can be mapped across the socio-ecological model but, while power may flow bidirectionally, it is clear that the power embedded throughout the model is weighted in a top-down direction in the context of gender-based violence in sport. Furthermore, sports unique context and its recognition as extended or surrogate family opens up a new avenue for those looking to understand and address gender-based violence in sport through the field of family violence.

There are limitations to our study. Most of the studies were conducted in high-income countries. The experience of athletes in middle- to low-income countries is therefore unknown. We included only English language publications, which means we may have missed studies from a broader range of countries. This compounds the limitation of the lack of diversity reported within the studies we reviewed. Future studies could address this by including and clearly documenting a more diverse sample of participants. The quality appraisal found some studies did not adequately describe their methods and methodologies. Future studies could pay greater attention to methodological rigor. Despite these limitations, the strength of our meta-synthesis lies in the synthesizing of first- and second-order constructs to go beyond describing the existing literature and establish an overarching interpretation of the collective of qualitative research on gender-based violence against women in sport.

There are several critical findings and implications arising from this review, which we summarize in Tables 4 and 5. However, the key takeaway from this review is that all those in sport, from sports scientists, health professionals, sports managers, to policymakers must understand that violence is experienced by women in many forms and often in composite. As such, initiatives to address such violence must be cognizant of violence in all its forms and the socio-ecological levels across which power and violence play out.

**Table 4.** Critical Findings.

• Women in sport often experience composite abuse, intersecting and overlapping, with power imbalance being at the heart of their experiences. • Women engage in safety work to deal with often expected gender-based violent behaviors in sport. • Power is a key factor relating to women’s experiences of gender-based violence and while women may be able to exert power through collective resistance, power remains with men and sports institutions that are complicit or actively engaging in violence. • Sport at the interpersonal and organizational level is a unique context that facilitates a quasi-family where family violence can occur.

**Table 5.** Implications for Research, Policy, and Practice.

• Given the diversity of gender-based violence experiences by women in sport, the lived experience voice must be central to any policy or practice development. • Policy and practice must attend to the inherent power imbalances across the socio-cultural, organizational, and interpersonal levels of the sport ecology in order to address gender-based violence experienced by women from individuals as well as institutions. • A gendered lens, cognizant of the five interacting socio-ecological levels in the sport context and the power embedded within and across them, is needed to guide development of appropriate initiatives to address gender-based violence in sport. • In recognition of the sport family, researchers and policymakers should look to the field of family violence prevention and response when exploring and developing appropriate initiatives. • Research must attend to the lack of diversity across study participants so that initiatives can be developed with the voices of all population groups participating in sport, including their intersections, for example LGBTQI, culturally and linguistically diverse communities, indigenous populations, and women with disabilities. • Qualitative research exploring women’s experiences of gender-based violence must ensure the methods and methodologies used are appropriate and adequately described in published works in order to provide confidence in the rigor of their work.

## Supplemental Material

sj-docx-1-tva-10.1177_15248380241244397 – Supplemental material for Women’s Experiences of Gender-Based Interpersonal Violence in Sport: A Qualitative Meta-Synthesis Supplemental material, sj-docx-1-tva-10.1177_15248380241244397 for Women’s Experiences of Gender-Based Interpersonal Violence in Sport: A Qualitative Meta-Synthesis by Kirsty Forsdike and Fiona Giles in Trauma, Violence, & Abuse

## Notes

1. Note this number includes 23 duplicates identified by Covidence that were not found during the Endnote duplicate identification process. These were primarily due to articles that were first published online before being published in journal editions and were present in both search 1 and search 2 records.
