# Supplementary material for: Women’s Experiences of Gender-Based Interpersonal Violence in Sport: A Qualitative Meta-Synthesis
Source: Trauma Violence Abuse. 2024 Apr 9;25(4):3254–68. doi: 10.1177/15248380241244397 (PMC11370166; doi:10.1177/15248380241244397)
Supplement: sj-docx-1-tva-10.1177_15248380241244397 – Supplemental material for Women’s Experiences of Gender-Based Interpersonal Violence in Sport: A Qualitative Meta-Synthesis [file sj-docx-1-tva-10.1177_15248380241244397.docx]

**Online Appendix A.** Example Search Strategy

Proquest

(noft(gender* N2 violenc* OR gender* N2 abuse* OR intimate partner N1 violence OR intimate partner N1 abus* OR domestic N1 violen* OR domestic N1 abus* OR sex* N1 abus* OR OR sex* N1 violen* OR dating violence OR sex* N1 harass* OR sex* N1 assault* OR coerc* OR rape OR battered wom?n OR non$accidental violence OR integrity) OR MESH(domestic violence OR coercion OR sexual abuse OR rape OR dating violence OR intimate partner violence OR gender based violence))

AND (noft(sport* OR physical* activ* OR leisur* OR active* recreation OR physical* recreation OR exercise* OR (group exercise OR group exercises) OR fitness OR gym* OR (fitness center OR fitness centers) OR (workout facilities OR workout facility)) OR MESH(Sports or physical activity OR leisure activities OR exercise OR group exercise OR fitness centers))

AND noft(qualitative* OR focus group OR focus groups OR focus-group* OR interview* OR ("life world" OR "life worldwide") OR ("lived experience" OR "lived experiences") OR ("participant observation") OR ethnograph* OR ("grounded theory") OR ("thematic analysis") OR hermeneutic* OR grounded-theor* OR phenomeno* OR interpretiv* OR interpretativ* OR constructiv* OR ("content analyses" OR "content analysis") OR "conversation analys*" OR ("mixed method" OR "mixed methods") OR experiences OR histories OR stories OR perception*)

AND noft(woman OR women OR female OR females)

**Online Appendix B.** CASP Quality Appraisal Checklist

| Study ID | Clear statement of aims of the research? | Qualitative methodology appropriate? | Research design appropriate to address  the aims of the research? | Recruitment strategy appropriate to the  aims of the research? | Data collected in a way that addressed  the research issue? | Relationship between researcher and  participants been adequately  considered? | Ethical issues been taken into  consideration? | Data analysis sufficiently rigorous? | Clear statement of finding? | How valuable is the research? |
| --- | --- | --- | --- | --- | --- | --- | --- | --- | --- | --- |
| Alexander 2020 | Y | Y | Y | Y | N | Y | N | N | Y | Y |
| Armstrong 2021 | U | Y | Y | Y | N | N | Y | N | Y | Y |
| Bisgaard 2019 | Y | Y | Y | U | Y | U | U | Y | N | Y |
| Brackenridge 1997 | U | Y | U | U | U | N | N | N | U | Y |
| Brockschmidt 2021 | Y | Y | Y | Y | Y | Y | U | Y | Y | Y |
| Fasting 2002 | Y | Y | Y | U | U | U | U | N | U | Y |
| Fasting 2007 | Y | Y | U | Y | Y | N | Y | U | Y | Y |
| Fasting 2009 | Y | Y | U | U | Y | N | Y | N | Y | U |
| Fasting 2015 | Y | Y | Y | Y | U | N | U | U | U | U |
| Hayden 2004 | Y | Y | U | Y | Y | U | Y | N | N | Y |
| Hussain 2021 | Y | Y | Y | Y | Y | Y | N | N | U | Y |
| Kirby 2000 | Y | Y | Y | U | U | N | U | U | Y | Y |
| Klavenes 2020 | Y | Y | Y | U | U | N | N | U | U | Y |
| Krauchek 1999 | Y | Y | Y | U | Y | Y | N | N | Y | Y |
| McMahon 2020 | Y | Y | Y | U | Y | U | Y | Y | Y | Y |
| McMahon 2021 | Y | Y | Y | U | Y | N | Y | Y | Y | Y |
| Owton 2016 | Y | Y | Y | Y | Y | Y | Y | U | Y | Y |
| Owton 2017 | Y | Y | Y | Y | Y | Y | Y | U | Y | Y |
| Rodriguez 2011 | Y | Y | Y | U | Y | Y | Y | U | Y | Y |
| Stirling 2009 | Y | Y | U | U | U | N | Y | N | U | Y |
| Tamminen 2013 | Y | Y | Y | Y | Y | Y | Y | Y | Y | Y |
| Tingle 2014 | Y | Y | Y | Y | Y | Y | U | Y | Y | Y |
| VanIngen 2020 | Y | Y | Y | U | U | Y | U | Y | U | Y |
| Vasudevan 2022 | N | Y | Y | Y | U | N | Y | Y | Y | Y |
| Way 2023 | Y | Y | Y | N | Y | U | N | U | N | Y |

Note. Y = Yes; U = unclear; N = Not.

**Online Appendix C.** Main characteristics of included studies (N=24)

| Study ID | Country in which the study was conducted | Participant role | Participant age | Sport type | Athlete diversity | No. of participants (no. of female participants) |
| --- | --- | --- | --- | --- | --- | --- |
| Alexander 2020 | Canada | Active or retired female Paralympic athletes | Not specified | Multiple individual sports | All participants had a disability. Ethnicity not specified | 8 (8) |
| Armstrong 2021 | New Zealand | Current athletes | Age range 18-45 years | Multiple sports (football, dancing, combat sports, cheer-leading, power lifting, race walking, triathlon, swimming, basketball, mountain biking, trail running, netball), some involved in multiple sports | Mainly NZ European, 2 Maori, 1 American, 1 North African | 20 (20) |
| Bisgaard 2019 | Denmark | 2 female athletes | Adults at time of interview; abuse occurred during childhood/ adolescence | One participant was a vaulter. The other athlete's sport was not specified. | Not specified | 2 (2) |
| Brackenridge 1997 | United Kingdom | Former female athletes | Not specified | Multiple sports (not specified) | Not specified | 11 personal accounts - 90 women in total sample |
| Brockschmidt 2021 | United Kingdom | Current male and female athletes | Age range 23-58 years (Average 32 years) | Running | Survey participant diversity not specified.  Interviews:  3 white, 1 white/Asian, 1 African Arab/Muslim, 1 white Jewish | Interviews: 9 (7) - Overall study 121 (69% female). |
| Fasting 2002 | Norway | Current female elite athletes | Age range 15-33 years (average 23 years) | 15 sports, 11 individual sports, 4 team sports | Not specified | 25(25) (2 excluded after interview) |
| Fasting 2007 | Norway | Former female elite Norwegian athletes (15 competed at Olympic level) | Age range 15 - 33 years (mean 23 years) | 15 different sports (16 competed in individual sports, 9 in team sports) | Not specified | 25 (25) |
| Fasting 2009 | Norway | Elite female athletes | Age range 15-33 years (average 23 years) | Represented 12 sports, 10 individual sports, 2 team sports | Not specified | 19 (19) |
| Fasting 2015 | Norway | Current and former female athletes | Aged 37 & 24 (harassment occurred during teenage years) | Handball, Volleyball | Not specified | 2(2) - Overall study 9(9) |
| Hayden 2004 | USA | Female college athletes who had been high school athletes. Athletes came from 7 colleges in USA.  Male and female high school coaches. Coaches came from all over USA. | Not specified | Multiple sports | Race/ethnicity  White (non-Hispanic) 89.9% for  athletes, and 93.5% for female coaches.  Black/African American  (3.6%) and Hispanic (2.9%) athletes.  Black/African American  Coaches (3.7%) | Athletes 138 (138) Coaches 156 (108) |
| Hussain 2021 | Pakistan | Sports managers | Not specified | Football | Pakistani | 2 (2) |
| Kirby 2000 | Canada | Current (75%) and retired (25%) male and female elite athletes | Age range 14-60 years (average 25 years) | Multiple sports | Race/ethnicity: Caucasian 85% other 15%. Disability status Yes 15% No 85% | 266 (146) |
| Klavenes 2020 | Spain | Sports administrators (from mid-level positions to management positions e.g., general managers, department directors) | Age range 24-48 years | Football | Not specified | 24 (12) |
| Krauchek 1999 | Canada | Current or former female varsity athletes | Age range 18-34 years | Soccer, swimming, track & field | All but one athlete was white | 32 (32) |
| McMahon 2020 | Australia | Former female elite athlete | Not specified | Swimming | Not specified | 1 (1) |
| McMahon 2021 | Australia | Former female elite athletes and club athletes | All <18yrs old at the time abuse occurred, but adults when interviewed | Swimming | Not specified | 3(3) |
| Owton 2016, 2017 | Unknown | Female athlete | Abused from the age of 13 to young adulthood (now mid-twenties) | Martial arts | Not specified | 1 (1) |
| Rodriguez 2011 | Puerto Rico | Former female athletes. Sporting experience ranged from 11-28 years (average 18 years) at various levels (school, collegiate, national, international); 4 had competed at elite level | Age range 31-54 years (average 43 years) | Not specified (for confidentiality) | 1 Black-Hispanic, 5 Hispanic-Puerto Rican | 6(6) |
| Stirling 2009 | Canada | Former female elite athletes (2-6 years retired) | Age range 21-26 years | 5 gymnasts, 5 swimmers | Not specified | 9 (9) |
| Tamminen 2013 | Canada | Female elite athletes - | Age range 18-23 years | 2 runners, 1 swimmer, 1 track & field, 1 basketball | 3 Caucasian, 2 'other' | 5 (5) |
| Tingle 2014 | United States of America | Female officials from 5 US states who officiated at high school and/or college level | Average age 37.25 years | Basketball | 7 white, 1 Latina | 8 (8) |
| VanIngen 2020 | Canada/United States | Former female professional athlete | n/a (article documents abuse over 20-year period using biographical research method) | Boxing | Not specified, however participant was identified in article and knowledge may have been assumed. | 1(1) |
| Vasudevan 2022 | USA, UK, Australia, Canada (meta-synthesis) | Females who participate in strength training | > 18 years | Strength training | Documented in systematic review | Multiple (14 papers included) |
| Way 2023 | United States of America | Former elite athletes, University athletes, and neighbours of the perpetrator | Not specified | Gymnastics | Not specified | 156 (156) |

**Online Appendix D.** Method and methodology of included studies (N=24)

| Study ID | Aim of study | Methodology | Theoretical framework | Researcher positioning | Method of recruitment | Method of data collection |
| --- | --- | --- | --- | --- | --- | --- |
| Alexander 2020 | To explore female Paralympic athlete views of effective and ineffective coaching practices | Collective case studies | Social constructionism | *Description* Previous experience working with athletes with a physical disability. *Reflection* Reflexive journal detailed first author's assumptions of coaching practices in parasport. Critical friend used to increase reflexivity and reduce bias. | Purposefully recruited by email | Semi-structured video or face-to-face interviews |
| Armstrong 2021 | To examine the framing of sport as a vehicle for positive change in relation to women's safety from men's violence | None reported | Empowerment as concept (Cattaneo and Goodman, 2015), Hargreaves (1994) feminist theories and Hartsock (1989) social constructionist approach. | *None reported* | Posters on Twitter and displayed in changing rooms of a University gym, as well as snowballing | Semi-structured interviews |
| Bisgaard 2019 | To examine how sexual harassment and abuse occurs and why it is difficult to terminate and report | Narrative inquiry | Bourdieu's field, capital and habitus, theory of action (as adapted by Hartill, 2017) | *None reported* | Part of a larger study (VOICE). 1 participant invited as known to researchers from previous study. The other was recruited via social media. | Unstructured interviews.  Key opening question: how would you describe your coach? |
| Brackenridge 1997 | To explain sexual abuse in sport, the female victim and the consequences of abuse for women and girls | None reported (described elsewhere Brackenridge 1996) | Finkelhor (1984) and Wolf (1984) models of abuse | *None reported* | Telephone help line established after a BBC television documentary program | Unstructured interviews, supplemented with information from publicly known cases and personal communications |
| Brockschmidt 2021 | To explore runners' experience of street harassment in London, the impact it has on runners and how they try to manage it. | Mixed method | Underpinned by interpretivism, adopting relativist ontology and a subjectivist epistemology. Followed Goffman as used by Greg Smith and Debra Gimlin. | *Description* First author is an 'insider' researcher. *Reflection* Research motivated by personal experiences & those of friends. Author wanted to understand and explain forms of street harassment and explore ways to prevent street harassment. Co-author acted as a 'critical friend'. | Recruited through social media, including Instagram and Facebook, as well as email and word of mouth. The first author contacted several ethnic minorities and lesbian, gay, bisexual, transgender, and queer (LGBTQ+) running clubs in London via email inviting them to take part. | Survey initially with follow-up interviews with 9 participants |
| Fasting 2002 | To establish an overview of sexual harassment incidence and to gather more information about risk factors, about elite athletes' reactions to sexual harassment, and about the consequences these experiences had for them | None reported | None reported | *None reported* | Identified via survey, and invited to participate in follow up interviews | Semi-structured interviews |
| Fasting 2007 | To understand the more immediate responses of the athletes to their harassment experiences | None reported | Vallerand and Blanchard (2000) | *Description*  The interviewer was an experienced female researcher and a former international athlete. | Initially completed survey. Then  received a letter asking if they would like to participate in an interview. | Semi-structured interviews (face-to-face) |
| Fasting 2009 | To examine 1) the usefulness of general classifications of perpetrators of sexual harassment and 2) whether qualitative data from sexually harassed elite female athletes support various conceptual frameworks for sexual harassment | None reported | Dziech and Wiener's (1984) and Pryor and Whelan's conceptual frameworks for sexual harassment | *None reported* | Initially completed survey. Then received a letter asking if they would like to participate in an interview. | Semi-structured interviews (face-to-face) |
| Fasting 2015 | To present two athletes' experiences of sexual harassment in narrative form and discuss how their stories can be explained and understood | Narrative analysis | multiple, mainly 4 factor theory (O'Hare and O'Donohue, 1998) and organisational model (Pina 2009) | *None reported* | Recruited from larger study – survey distributed to sport and PE students, followed by 9 semi-structured interviews with Norwegian female athletes who reported sexual harassment. Two narratives selected. | Semi-structured interviews |
| Hayden 2004 | To assess 1) perceptions of athletes and coaches regarding sexual harassment by male and female high school coaches; 2) differences/ similarities in the perceptions of female athletes, female coaches and male coaches regarding sexual harassment; 3) incidence of sexual harassment by high school athletic coaches | None reported | None reported | *None reported* | Convenience sampling. Author (Doctoral student) had existing contacts with various associations and colleges. | Paper-based survey (with supplementary open-ended questions). Coaches survey distributed via mail to identified potential participants. Athlete survey distributed by College and University Athletic Directors. |
| Hussain 2021 | To explore the lived experiences of two female sports managers in Pakistan | Co-constructed narratives | Intersectionality (McCall, 2005) | *Description* First author practicing Muslim from Pakistan. Second author white Christian American scholar. *Reflection*  First author kept a reflective journal in which he sought to recognise women's marginalisation in a patriarchal system. | First author made initial contact with participants. Unclear how they were identified initially. | Interviews |
| Kirby 2000 | To describe and explain sexual abuse in sport in Canada, and propose a way forward to create a more ethical and caring sporting climate | None reported | None reported | *Description* All are researchers with sociology, health and political scientist backgrounds. Not directly involved in sport/sporting administration. | None reported | Cross-sectional survey with qualitative and quantitative components |
| Klavenes 2020 | To provide more knowledge about the impact of the #MeToo phenomenon in football; and observe the impact gender roles have on women's professional careers in the football industry | None reported | Risman's theory of gender a social structure (2004) | *None reported* | Direct contact by researchers and horizontal networking approach (snowballing) | Semi-structured telephone or face-to-face interviews |
| Krauchek 1999 | To explore the experiences and perceptions of sexual harassment among women athletes coached by men in elite sport | Instrumental case study | Radical feminist perspective (mainly Hall 1995). Sexual harassment permeable, open to multiple interpretations and resistance | *Description* First author an athlete who had competed at provincial, national and international levels. Her sporting career had involved relationships with male coaches.  *Reflection* Experience informed the interview guide, and shaped discourse between interviewer and respondent | Not reported. However, authors refer to one Western Canadian University were initial contacts with some team members were made then led to a snowball sample | Semi-structured interviews |
| McMahon 2020 | To bring forward the emotional, evocative, lived experiences of one athlete | Storytelling grounded in narrative inquiry | None reported | *None reported* | None reported | Interview (face-to-face) |
| McMahon 2021 | To look at one way (body shaming) psychological abuse may be enacted and the role the athlete entourage plays | Storytelling | Social constructionism | *Description* Author experienced abuse in swimming. Also aware of power differentials experienced by athletes in sport and did not want to replicate that in the study. *Reflection* Choice of storytelling as methodological approach to address these concerns | Not reported | 'Emotional recall' strategy, followed by semi-structured interviews (face-to-face) |
| Owton 2016, 2017 | To explore the abuse of power and the dynamics of abuse between the athlete (the victim) and the perpetrator (coach), including the grooming process | Collaborative autoethnography | Feminist ethics of care | *Description* Researcher knew participant as friend through shared involvements in similar sports. Participant approached researcher to become involved in study. *Reflection* Stance of friendship (Allen-Collinson 2012). Second author acted as 'critical friend'. | Participant known to researcher, participant self-identified to be a part of the study | Poems and vignettes |
| Rodriguez 2011 | To explore Puerto Rican female athletes' experiences and coping strategies with sexual harassment situations within the context of sport | Phenomenology | Interpretive framework | *Description* Former elite athlete and professional career in sport. *Reflection*  Used technique of bracketing (Moustakas, 1994) - researchers set aside experiences to take a fresh perspective. | Authors presented at a national conference; participants expressed interest in sharing experiences | Semi-structured interviews |
| Stirling 2009 | To investigate abused athletes' perceptions of the power held by coaches, and the influence this had on their experiences of abuse | None reported | None reported | *None reported* | Snowball sampling starting with one abused athlete, researchers contacted potential participants by telephone | Semi-structured interview (face-to-face) |
| Tamminen 2013 | To:  - explore experiences of adversity and  - examine perceptions of growth following adversity among elite female athletes | Interpretative Phenomenological Analysis | None reported | *Description* Researchers reported they had no elite athlete experience nor had experienced adversity. *Reflection D*iscussed positioning from the perspective of bracketing (Allen-Collinson, 2009) and participants as teachers (Flick, 2002) | University coaches were contacted to inform their athletes about the study. Coaches distributed information to their athletes who then contacted the researchers to participate in the study. | Semi-structured interviews (face-to-face) |
| Tingle 2014 | To examine the experiences of former female basketball officials to understand the shortage of female sport officials | Phenomenology | Workplace incivility framework | *Description* One researcher had experience as a basketball official. *Reflection D*escribed from an 'insider/outsider' perspective (Allen-Collinson, 2009) and 'bracketing' (Willig, 2007; Ashworth, 1996). | Snowballing. Emails initially sent to officials who administer lists of former officials who were emailed and asked if they would like to participate. They were in turn asked for contact information for other eligible participants. | Semi-structured interviews |
| VanIngen 2020 | To highlight the ways in which boxing functions both as a site of and a sanctuary from gender-based violence | Biographical narrative, rooted in sociological imagination | None reported | *Description* Amateur boxer and travelled with professional boxer. Set up boxing program for female survivors of violence. *Reflection D*iscusses importance of locating social position of author (Letherby, 2018; Stanley, 1993). | None reported | Media sources, transcripts from criminal trial, interview (face-to-face) |
| Vasudevan 2022 | To describe the barriers and motivators for strength training in adult women | Systematic review and meta-synthesis |  | *None reported* | N/A | Searched PsychINFO, PubMed, ASSIA |
| Way 2021 | To discover what organizational structures kept girls participating in gymnastics even as it became an obstacle to their flourishing. | None reported | Berlant's affective theory of "cruel optimism" (2011), Bessant's "opaque violence" (1998) and Harris's framing of sexual violence as an organisational phenomenon (2019) | *None reported* | N/A (secondary data in the public domain) | Video footage obtained from YouTube.com and written transcripts obtained from the “InOurOwnWords.us” website. The transcripts were uploaded into NVivo for coding. |
